# Fear, opposition, ambivalence, and omission: Results from a follow-up study on unmet need for family planning in Ghana

**DOI:** 10.1371/journal.pone.0182076

**Published:** 2017-07-31

**Authors:** Sarah Staveteig

**Affiliations:** 1 Avenir Health, Glastonbury, Connecticut, United States of America; 2 The Demographic and Health Surveys (DHS) Program, Rockville, Maryland, United States of America; Iranian Institute for Health Sciences Research, ISLAMIC REPUBLIC OF IRAN

## Abstract

**Introduction:**

Despite a relatively strong family planning program and regionally modest levels of fertility, Ghana recorded one of the highest levels of unmet need for family planning on the African continent in 2008. Unmet need for family planning is a composite measure based on apparent contradictions between women’s reproductive preferences and practices. Women who want to space or limit births but are not using contraception are considered to have an unmet need for family planning. The study sought to understand the reasons behind high levels of unmet need for family planning in Ghana.

**Methods:**

A mixed methods follow-up study was embedded within the stratified, two-stage cluster sample of the 2014 Ghana Demographic and Health Survey (GDHS). Women in 13 survey clusters who were identified as having unmet need, along with a reference group of current family planning users, were approached to be reinterviewed within an average of three weeks from their GDHS interview. Follow-up respondents were asked a combination of closed- and open-ended questions about fertility preferences and contraceptive use. Closed-ended responses were compared against the original survey; transcripts were thematically coded and analyzed using qualitative analysis software.

**Results:**

Among fecund women identified by the 2014 GDHS as having unmet need, follow-up interviews revealed substantial underreporting of method use, particularly traditional methods. Complete postpartum abstinence was sometimes the intended method of family planning but was overlooked during questions about method use. Other respondents classified as having unmet need had ambivalent fertility preferences. In several cases, respondents expressed revised fertility preferences upon follow-up that would have made them ineligible for inclusion in the unmet need category. The reference group of family planning users also expressed unstable fertility preferences. Aversion to modern method use was generally more substantial than reported in the GDHS, particularly the risk of menstrual side effects, personal or partner opposition to family planning, and religious opposition to contraception.

## Introduction

Unmet need is a central concept in family planning research and a key indicator for gauging the demand for contraception and for measuring the success of programs and policies. At its most basic level, unmet need reflects an apparent discrepancy between women’s stated reproductive preferences and behavior. In surveys some women respond they want to space or limit births, but they are not using any method to prevent pregnancy. These respondents are considered to have unmet need for family planning, and are at risk of unintended pregnancy. Unintended pregnancies frequently lead to unsafe abortions or maternal complications and place the health of mothers and children at risk [1].

In 2012 there were estimated to be more than 74 million unintended pregnancies in the developing world, including 4.6 million in West Africa [2]. The global burden of unintended pregnancy includes not only health of mothers and their children, but also costs for health care systems, social costs, and economic well-being of families worldwide. Family planning provides women and families the opportunity to plan and space births in line with their own reproductive preferences, helping to ensure the safety and well-being of both mother and children. Monitoring unmet need has taken on increased emphasis in recent years as policymakers seek to help women and couples achieve their reproductive goals. Reducing unmet need was part of the Millennium Development Goals. An indicator derived from unmet need, demand satisfied for modern contraception—computed as modern contraceptive prevalence divided by the sum of unmet need for modern contraceptive methods and modern contraceptive prevalence—is an indicator for the Sustainable Development Goals and a central part of new efforts by USAID and several donors to scale up family planning for millions of women as part of FP2020 [3].

The Demographic and Health Surveys (DHS) Program, the largest source of data on contraceptive patterns and unmet need in developing countries, conducts nationally representative surveys during which interviewers ask women questions about sexual activity, fertility preferences, fecundity, contraceptive use, and other topics. DHS and other nationally representative surveys, such as Multiple Indicator Cluster Surveys (MICS) and Performance Monitoring and Accountability 2020 (PMA 2020), compute unmet need for family planning based on a complex algorithm involving women’s responses to 18 questions asked at various points throughout the interview [4]. Married women, and in some cases sexually active unmarried women, who are fecund and wish to postpone giving birth for two or more years or stop childbearing altogether but who are not using any method of family planning are classified as having unmet need. Additionally, women who are pregnant or postpartum amenorrheic with an unwanted or mistimed pregnancy are considered to have an unmet need for family planning.

Specifically, women are considered to have unmet need if they are in any of the following three categories: (1) at risk of becoming pregnant, not using contraception, and want no more children, or want children but do not want to become pregnant within the next two years, or are unsure if or when they want to become pregnant; (2) pregnant with a mistimed or unwanted pregnancy; or (3) postpartum amenorrheic for up to two years following an unwanted or mistimed birth and not using contraception [4]. The calculation of unmet need does not involve direct questions about women’s own contraceptive preferences and proclivities; as such, it is described as a measure of latent or potential demand for family planning [5, 6].

Relatively few women with an unmet need for family planning in developing countries cite cost or access as reasons for not using a contraceptive method [7]. Instead, survey respondents tend to cite fear of side effects, abstinence, breastfeeding, and attitudinal factors. The explanations underlying these stated reasons are not well understood.

The mixed methods follow-up study described in the present article leveraged the existing sampling structure and data collection within the 2014 Ghana Demographic and Health Survey (GDHS) to provide additional insights into reproductive preferences and barriers to family planning among women in Ghana with unmet need. The primary objective of the follow-up study was to better understand the lived experience and meanings underlying the apparent contradiction in fertility preferences and reproductive behavior that produce statistical estimates of unmet need in the DHS surveys. Do the survey questions about current family planning use and reproductive preferences retain their intended meaning in the field? How stable and well defined are women’s fertility preferences, and how do women explain their non-use of family planning? The study compares respondents classified as having unmet need with a reference group of respondents who were using family planning at the time of the survey.

Open-ended, qualitative questions can provide substantial insight into ambivalence, perceptions, and attitudes not readily apparent from large-scale survey data. Qualitative and mixed methods studies are well positioned to provide important insights about demographic behaviors [8, 9] and are particularly relevant to understanding the meanings that respondents attach to responses in large-scale surveys such as the DHS [10].

### The concept of unmet need

The development of the concept of unmet need is rooted in the KAP (Knowledge, Attitude, and Practice) surveys of the 1960s. Researchers identified married women whose preferences and behavior appeared contradictory—that is, they wanted to limit or space childbearing but were not using a method of family planning [11]. The KAP surveys gave way to the World Fertility Survey program in the 1970s and 1980s and ultimately evolved into the Demographic and Health Surveys (DHS) Program which covered a wider range of topics, starting in 1984. The algorithm for determining unmet need grew increasingly complex over time as questions on fertility preferences evolved and data from the contraceptive calendar were included when available. In 2012, a simplified, consensus DHS/MICS definition of unmet need was established using a standard algorithm [4]. A comprehensive history of unmet need and evolution of the classification schema has been detailed by other authors [12–14].

One concern with the concept of unmet need is that the term itself implies a demand for family planning—and, in fact, is summed with contraceptive prevalence to compute an indicator called “demand for family planning”—but the term does not necessarily reflect actual or potential interest in method use. In particular, it does not reflect how women themselves perceive their risk of pregnancy, the strength of their preferences, or their interest in or resistance to family planning. Additional concerns about the measurement of unmet need include the failure to differentiate married women who are sexually active from those who are not, and thus at no risk of pregnancy [12], the failure to include male partners [15, 16], and instability in professed fertility preferences [17–20].

The extent to which survey measures of unmet need gauge latent demand for family planning has been questioned due to the temporal instability of the measure and the number of different groups it encompasses [21]. Even in the early days of the development of unmet need as a measure of demand for family planning, it was known that in some countries less than half of women with unmet need were currently at risk of pregnancy [14]; unmet need classification depends on prospective fertility preferences as well as on ex-post facto assessments of the intendedness of pregnancies and recent births. A technical working group for FP2020 opted against using reductions in levels of unmet need as a global goal because it is not a unidirectional measure of programmatic success [22].

Despite concerns about its measurement and interpretation, unmet need is a powerful concept. Abortions, surreptitious use of family planning, and unwanted pregnancies all attest to an ongoing need for family planning that is unfulfilled [23]. How to assess women’s ‘need’ or demand for family planning has proven difficult, however. In particular, evidence indicates that—even among survey respondents whose fertility operates within the “calculus of conscious choice”—the answers to prospective fertility preference questions are fraught with ambiguity and uncertainty [17, 19]. Measures of unmet need depend on women’s tendency to plan and articulate fertility preferences in a two-year window. Fertility preferences are subject to both social context [5] and to vital conjunctures in women’s lives [24], including husband’s desire for children [16, 25], future economic well-being, marital stability, and survival of current children. Longitudinal evidence finds substantial instability in individual women’s fertility preferences over time [26–28].

### Study context

At the time this study was being designed, Ghana had recorded one of the highest levels of unmet need for family planning among married women on the African continent, at 36 percent in 2008 [29]. Family planning use had declined slightly among married women, from 25 percent in 2003 to 24 percent in 2008. Meanwhile, Ghana’s Total Fertility Rate (TFR) in 2008 was among the lowest in West Africa, at 4.0 births per woman. Unmet need is typically only measured among currently married women, but the focus of this study is currently married and sexually active women combined, as both groups of women are at risk of unwanted pregnancies. Nationwide, 29 percent of married and sexually active unmarried women in Ghana had an unmet need for family planning as measured by data from the 2014 GDHS.

Ghana’s attainment of regionally low fertility despite modest levels of family planning use has been a demographic puzzle for nearly two decades [30]. Abortion is legal in Ghana and has been hypothesized as a reason for lower-than-expected fertility [31]; but evidence has been inconclusive. It may be that high levels of unmet need in Ghana partly reflected women’s growing tendency to articulate a need for spacing or limiting births. During early stages of the demographic transition, the percentage of women with unmet need can increase even as demand for family planning is being satisfied simply due to women’s increased interest in reducing fertility [32].

Ghana has a relatively strong family planning program. The contraceptive method mix is diverse. Injectables, the pill, and implants are the most common methods, followed by the rhythm method. Women can obtain contraception from public and private sources. Family planning is inexpensive but not free. Ghana does experience occasional contraceptive supply issues and there are some limits to the method mix offered. Social marketing campaigns have proven successful but some very remote areas of the country remain a few hours’ distance from the nearest clinic. Even so, in surveys women rarely cite access and cost as reasons for non-use of family planning [33].

By 2014 the TFR increased slightly, to 4.2 births per woman [34]. The 2014 GDHS also found an increase in modern contraceptive prevalence since 2008 (from 17 to 22 percent) and a decline in unmet need (from 36 to 30 percent) among married women ages 15–49. This brings the country on par with levels of unmet need in neighboring West African countries, but still high in a global perspective.

Perceptions about side effects and attitudinal factors pose a challenge to increased family planning use in Ghana. Focus group discussions from a hospital in Ghana found women’s concern with menstrual regularity results in dissatisfaction with family planning methods that prevent menstruation [35]. Another study found that many Ghanaian women perceive family planning as ineffective or unsafe [36], and DHS data from 1988 to 2008 show that attitudinal resistance has been an increasing component of unmet need in Ghana [33]. Male attitudes toward contraception are mixed: in the 2014 GDHS, 73 percent of men age 15–59 rejected the idea that contraception is a woman’s business and men should not have to be involved, but 46 percent supported the statement that women who use contraception may become promiscuous [34]. Married Ghanaian women’s sexual empowerment is a statistically significant predictor of contraceptive use, even after controlling for other factors [37].

## Methods

This study was designed as a data-linked embedded follow-up study, of the type described by Schatz [38, 39]. It was independently funded, planned, and fielded, but respondents were systematically selected from among the respondents to the 2014 GDHS. The 2014 GDHS is a nationally representative household survey in which 9,396 women age 15–49 were interviewed [34]. Fieldwork for the GDHS was conducted by the Ghana Statistical Service (GSS) and the Ghana Health Service (GHS), with technical assistance from ICF International through The DHS Program, which is funded by USAID. At the end of the GDHS, all 9,396 female respondents were asked for consent to be re-contacted for a follow-up study on family planning. Nationwide, 99.6 percent of women agreed to be re-contacted.

The follow-up study selected three of ten regions for follow-up: Greater Accra, the capital, Central Region, and Northern Region. Central Region had the highest level of unmet need in 2008 and Northern Region has typically had higher fertility and low family planning use. In all, 13 survey clusters were selected for follow-up fieldwork five in Northern Region, five in Central Region, and three in Greater Accra, the region with the lowest fertility. It was decided in advance that all three clusters selected in Greater Accra would be urban, and that one of the five clusters in Central Region and in Northern Region would be urban. A completely random selection of clusters from the parent survey would not have been feasible. Fieldwork for the GDHS takes four months, versus one month for the follow-up study, and it was desirable to return within three weeks. Additionally, for a small-scale study the distances might have been prohibitive. Thus cluster selection was based on those available at the time of the study, with an eye toward geographic diversity. However, within a given cluster women were selected systematically, from among those surveyed in a stratified random sample rather than a convenience sample typical for qualitative studies.

Fieldwork for the follow-up study was conducted by the Institute for Statistical, Social, and Economic Research (ISSER) at the University of Ghana, Legon. Preparation for interviews began with an 11-day training and pretest in Accra. Along with a guide from GSS, three field teams each consisting of two interviewers and a field supervisor attempted to relocate the selected DHS respondents within three weeks of the original survey. Interviewers returned up to three times to complete the interview. Interviews were randomly audited to ensure that they were correctly completed. Follow-up interviews were conducted anywhere from 5 to 60 days after the GDHS interview; on average women were reinterviewed 20 days after their GDHS interview. Survey procedures are described in detail elsewhere [40, 41].

**Fig 1:**
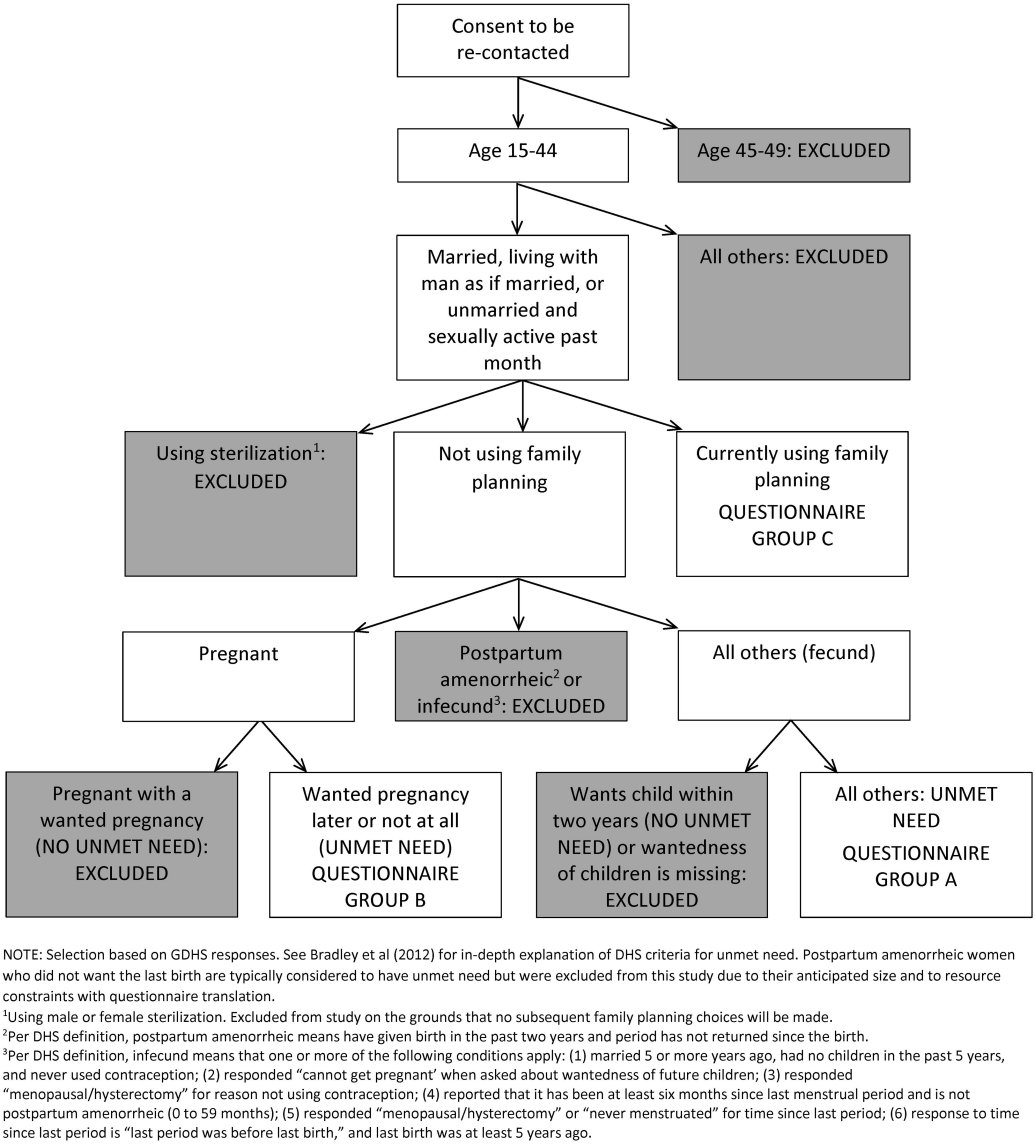
Sample selection.

The overall response rate was 92.3 percent (131 of 142 women selected). Of these 131 respondents, 96 qualified for analysis. Fig 1 shows detailed sample selection criteria. This article analyzes 96 respondents, the 50 women who were classified by the GDHS as having an unmet need for family planning, alongside a reference group of 46 women who were identified as having a met need for family planning.

Respondents to the follow-up survey were located by address, name of household head, and relationship to the household head; to verify their identity, respondents were asked six additional questions: year of birth, month of birth, marital status, whether ever given birth, number of resident sons, and number of resident daughters. The vast majority of respondents matched on all or all but one characteristic. The 96 cases discussed here reflect confident—but imperfect—identity matching. Prior studies have found inconsistent results on key questions, even with the exact same survey conducted after only a short delay [42, 43]. Discrepancies in responses were flagged so that interviewers could inquire further.

Follow-up interviews were conducted using Android tablets to import respondent data and guide questions; audio recorders were used to capture women’s full responses to each question. Questionnaires were translated into three languages—Twi, Ga, and Hausa. The semi-structured questionnaires included a combination of closed and open-ended questions about reproductive preferences, ambivalence, decision-making, and family planning (see S1 File). Respondents were also asked open-ended questions about fertility desires, family planning use, attitudes toward family planning, role of partner and extended family in decision-making, and barriers to access.

After fieldwork was complete, audio files from the interviews were transcribed into the language of the interview and subsequently translated into English, resulting in over 1,000 single-spaced pages of transcripts. Transcripts were input into ATLAS.ti qualitative analysis software. A number of themes were established and listed at the start, based on the questionnaire, while additional themes were added inductively by iteratively reading transcripts. A list of themes was developed, refined, and independently applied to a set of test transcripts by two raters to compare reliability. After finalizing the schema, the themes were consistently applied to the transcripts in ATLAS.ti. Variables created directly from tablet entry information were reviewed for missing and inconsistent values and, when possible, filled in or manually verified against transcripts. These data were confidentially linked to publicly available GDHS records and analyzed using Stata.

### Ethical considerations

The ICF International Institutional Review Board (IRB), which requires compliance with the U.S. Department of Health and Human Services regulations for the protection of human subjects (45 CFR 46), reviewed and approved all study procedures and questionnaires. A waiver of written consent was obtained from the IRB due to minimal risk of harm and a lack of procedures for which written consent is normally required. Respondents were asked for verbal consent to be re-contacted during the main survey and for verbal consent to be interviewed and to be audiotaped at the start of the follow-up interview. Before the interview began interviewers were required to provide their electronic signature attesting that they had received verbal consent from the respondent to be interviewed and that they had correctly indicated whether the respondent consented to be audiotaped.

In keeping with IRB regulations and The DHS Program’s practices, the confidentiality of the respondent’s information was maintained at all stages of the survey. Recordkeeping used anonymous cluster and respondent identifiers. As voluntary HIV serotesting was conducted during the 2014 Ghana DHS, no data entry on names or addresses was done and all cluster and household numbers were scrambled prior to linkage with HIV test results. Similarly, at the conclusion of fieldwork the implementing agency for the follow-up study destroyed all identifying information used to contact respondents, maintaining only an anonymized identification number. A linkage between the anonymized identification number for the follow-up study and the final, publicly available dataset is kept only by The DHS Program.

## Results

### Sample characteristics

The 96 married and sexually active unmarried women analyzed in this article were systematically selected from among GDHS respondents. The follow-up sample was intended to be diverse, but given the scale of the study, the sample was not designed to be perfectly representative of the three regions. Table 1 indicates how the characteristics of the sample as defined in the GDHS compared with family planning users and with women who had an unmet need in the three selected regions. GDHS sample weights are applied to regional percentages and to the sub-study respondents. Table 1 shows that respondents to the follow-up study were more concentrated in their 30s than their respective regional counterparts. Fewer follow-up respondents were age 15–19. Follow-up respondents were more predominantly rural than both family planning users and women with unmet need in the country as a whole.

**Table 1:** Background demographic characteristics of follow-up respondents compared with married and sexually active unmarried women in three selected regions, GDHS 2014.

Percentage distribution of background characteristics within the following groups:				
	Family planning users in three study regions^1^	Follow-up family planning users^2^	Women with unmet need in three study regions^1^	Follow-up respondents with unmet need^2^
**Age**				
15–19	3	2	10	4
20–24	17	20	17	18
25–29	26	28	20	12
30–34	22	22	18	26
35–39	14	20	21	22
40–44	12	9	9	18
45–49	7	▪	4	▪
**Residence**				
Urban	61	39	60	26
Rural	39	61	40	74
**Region**				
Central	33	41	27	32
Greater Accra	56	28	49	14
Northern	11	30	24	54
**Education**				
No education	15	20	27	46
Primary education	16	11	17	16
Secondary and above	69	70	56	38
**Religion**				
Catholic	8	20	6	6
Anglican	1	0	2	0
Methodist	8	7	5	4
Presbyterian	7	7	4	4
Pentecostal/Charismatic	41	37	44	32
Other Christian	20	11	13	14
Islam	11	11	20	20
Traditional/Spiritualist	2	4	4	10
No religion	2	4	3	10
Other	0	0	0	0
**Ethnicity**				
Akan	52	57	41	34
Ga/Dangme	14	4	14	6
Ewe	14	7	12	6
Guan	1	0	3	0
Mole-Dagbani	9	9	16	18
Grusi	2	2	1	0
Gurma	5	22	9	36
Mande	0	0	0	0
Other	3	0	3	0
Total	100	100	100	100
**Number of women**	**625**	**46**	**746**	**50**

Note: Percentages may not sum to total due to rounding.

▪ Zero due to sampling criteria

^1^ National and regional estimates are weighted.

^2^ Follow-up respondents are shown unweighted for ease of comparison with case count tables.

Women who reported using family planning in the GDHS were more highly educated and wealthier than women with unmet need. Both follow-up samples were over-representative of the lowest wealth quintile than the regional averages. The follow-up sample exhibited religious diversity, but was more heavily traditional/spiritualist and no stated religion than women nationwide in both the unmet need and family planning groups. One of the clusters in the North was Konkomba-speaking, and respondents were ethnically Gurma; the follow-up study sample was thus much more heavily comprised of Gurma women than the country as a whole.

Reproductive characteristics of the four groups (regional and follow-up respondents who were family planning users and who had unmet need in the GDHS) are shown in Table 2. Women’s knowledge of family planning methods is high in Ghana. Nationwide, over 99 percent of women with unmet need know at least one modern method. In the follow-up survey, one respondent was identified by the GDHS as not knowing any method of family planning, another as only knowing traditional methods. The majority of women with unmet need have used a family planning method before.

**Table 2:** Background reproductive characteristics of follow-up respondents compared with married and sexually active unmarried women in three selected regions, GDHS 2014.

Percentage distribution of background characteristics within the following groups:				
	Family planning users in three study regions^1^	Follow-up family planning users^2^	Women with unmet need in three study regions^1^	Follow-up respondents with unmet need^2^
**Marital Status**				
Never in union	14	17	17	14
Currently in union	83	76	80	84
Formerly in union	4	7	4	2
**Number of children ever born**				
0	17	13	14	14
1–2	31	41	34	20
3–5	40	39	38	36
6+	12	7	14	30
**Knowledge of family planning**				
Knows no method	▪	▪	1	2
Knows only traditional method	0	0	1	2
Knows modern method	100	100	99	96
**Unmet need**				
Unmet need for spacing	▪	▪	69	62
Unmet need for limiting	▪	▪	31	38
Using for spacing	61	78	▪	▪
Using for limiting	39	22	▪	▪
**Ever use of family planning**				
Ever	100	100	62	66
Never	▪	▪	39	34
**Abortion experience**^3^				
Never had an abortion	-	72	-	90
Had an abortion once	-	24	-	6
Had an abortion more than once	-	4	-	4
Total	100	100	100	100
**Number of women**	**625**	**46**	**746**	**50**

Note: Percentages may not sum to total due to rounding.

▪ Zero by definition or due to sampling criteria

^1^ National and regional estimates are weighted.

^2^ Follow-up respondents are shown unweighted for ease of comparison with case count tables.

^3^ Abortion not asked about in GDHS; results from follow-up study only

### Fertility preferences

Fertility preferences are a pivotal component of unmet need. Among fecund women, declared intention to have a/another birth and the preferred timing of the next birth determine unmet need status. The two questions on reproductive preferences used to compute unmet need for this group are: (1) “*Would you like to have (a/another) child*, *or would you prefer not to have any (more) children*?*”* Allowable responses to this question are: (a) want a/another; (b) no more; (c) cannot get pregnant; (d) a special condition, such as ‘after marriage’; or (e) don’t know/undecided. Non-pregnant respondents who want a/another child are then asked: (2) *“How long would you like to wait from now before the birth of (a/another) child*?*”* Pregnant women are asked: *“After the child you are expecting now*, *would you like to have another child*, *or would you prefer not to have any more children*?*”* The answer must either be a specific number of years and months, a special wait condition, or undecided. While the questions used to ascertain fertility preferences are seemingly straightforward, women’s ability and willingness to articulate a fixed timeline for their preferred time to next birth are culturally and temporally variable.

Non-pregnant respondents who want no more children and meet criteria for fecundity and non-use are considered to have an unmet need for limiting. The consensus definition of unmet need uses a threshold of two or more years to determine whether women have an unmet need for spacing. A comparison between responses to the question from the GDHS and the follow-up survey are shown in Table 3. As the table indicates, 18 percent of follow-up respondents with unmet need and 13 percent of follow-up family planning users gave inconsistent answers to this question between surveys. The shift in responses occurred largely among women who stated they were undecided in the GDHS but said they wanted another child upon follow-up. There was some additional negligible movement in both directions between wanting no more children and wanting another child during the follow-up.

**Table 3:** Comparison of preference for a/another child as reported to the GDHS and in follow-up survey.

*Panel A*: *Follow-up respondents with unmet need*					
	Follow-up: Have a/another	Follow-up: Undecided	Follow-up: No more	Total	Discrepant
GDHS: Have a/another^1^	23	0	1	24	4%
GDHS: Undecided	6	1	0	7	86%
GDHS: No more	2	0	17	19	11%
**Total**	**31**	**1**	**18**	**50**	18%
*Panel B*: *Follow-up family planning users*					
	Follow-up: Have a/another	Follow-up: Undecided	Follow-up: No more	Total	Discrepant
GDHS: Have a/another	33	0	1	34	3%
GDHS: Undecided	2	0	0	2	100%
GDHS: No more	3	0	7	10	30%
**Total**	**38**	**0**	**8**	**46**	13%

^1^ Non-pregnant women are asked: *"Now I have some questions about the future*. *Would you like to have (a/another) child*, *or would you prefer not to have any (more) children*?*" Pregnant women are asked*: *"After the birth of the child you are expecting now*, *would you like to have a/another child or would you prefer not to have any more children*?*"*

The main ambivalence in fertility preferences that emerged through interviews was not whether to have another child, but the timing of that preference. In the GDHS, responses to question about desired timing of the next birth were limited to a single number of months or years, or to a special wait condition. In the follow-up survey women were allowed to specify an open range of desired time until the next birth. Responses to this question, grouped by minimum wait time into one-year intervals, are shown in Table 4 for follow-up respondents with unmet need. Figures include the eight respondents who said in the GDHS that they were undecided or that they wanted no more children but declared in the follow-up survey that they wanted a/another. Twenty of 31 respondents indicated a desire to wait until a single point in time, while the remainder gave a time range, averaging 23 months.

**Table 4:** Preferred minimum waiting time to next birth among follow-up respondents with unmet need, according to follow-up survey.

Among 31 follow-up respondents with unmet need who want a/another child, response to the question in follow-up study *"(After the birth of the child you are expecting now / How long would you like to wait from now) before the birth of (a/another) child*?*"*					
Minimum wait time (grouped)	Fixed	Range^1^	Total	Average strength of desire to delay that long^2^	Would affect unmet need designation?
Soon, now	3	-	**3**	-	Yes
1 to 11 months	1	2	**3**	4.3	Yes, if fixed at <2 years
12 to 23 months	3	2	**5**	3.8	Yes, if fixed at <2 years
24 to 35 months	4	1	**5**	3.2	No
36 to 47 months	2	3	**5**	2.6	No
48 to 59 months	1	1	**2**	5.0	No
60 to 71 months	4	1	**5**	4.2	No
72 months and beyond	1	1	**2**	4.5	No
Don’t know	1	-	**1**	-	No
	**20**	**11**	**31**		

^1^ Average width of range is 23 months.

^2^ Response to question *"Now*, *please tell me how strongly you feel about waiting that long to get pregnant*. *Please give me a number between 0 and 5*, *where 0 means you do not mind becoming pregnant before the time you stated and 5 means you* *really* *want to avoid getting pregnant before that time*.*"* Response does not apply to women who wanted a birth without delay (soon or now). The average strength of "no more" (for 18 respondents not listed here) is 3.6.

Of the respondents classified as having unmet need who indicated in the follow-up interview that they wanted a/another birth, 11 declared in follow-up a minimum desired waiting time of less than two years. For seven of these 11 respondents, two years encompassed both the minimum and maximum waiting time, which would have classified them as having no unmet need. The other four respondents gave a range of time that started before two years. If they had been asked to give a fixed number and settled on less than two years, they would also have been excluded from the group defined as having unmet need.

The follow-up survey also asked women the strength of their desire to wait that long for the next birth. Results are shown in Table 4. Desires to delay were weakest among women who expressed a preference to wait one to three years; they tended to be strongest at the highest and lowest time boundaries.

A main theme that emerged from the discussion of fertility preferences overall was that respondents felt torn between joy versus means—or in many cases torn between the potential future value of a child (who could be the “star of the family”) versus the immediate cost of raising a child. Respondents and their partners valued children highly, but most respondents perceived an inherent trade-off between the value of another child and the financial or health costs of too many or closely spaced births. For example, as a 35-year old woman in urban Central Region (R03.08) described:

*Well, it would be good to have another child, and it would be especially good to have a safe delivery. Even the Bible tells us that children are a blessing and a gift from God, so if I have another child and am able to take care of him or her such that he grows to become responsible and respectable person, it would even bring honor to me. People would point at him and say, oh there goes Sister [Name]’s son, and that would bring me fulfillment and joy… I do not know how far the child might go in life; he might even be an important personality and bring honor to our family, so that could be a value that having lots of children might bring us… Even if you look at the Bible, it says to be fruitful and multiply and to replenish the earth, it’s out of many children that some grow to be important personalities of the world but it’s due to economic and financial hardships that people face that’s why they may decide to have fewer children but ideally, you should have more, you never know which of your children would be of significance someday*.

Respondents frequently indicated that they would accept an unwanted pregnancy but would not be happy. “*I don’t like it but if it comes I will accept it”* or “*I will not be happy but once it has come I will accept it”* was how seven respondents (R01.01, R01.03, R01.05, R02.03, R02.07, R04.02, R05.07) described their attitude toward having another child. *“I will be disturbed but I will be happy to have a child”* said another (R01.07).

For the 11 respondents identified as having an unmet need whose timing shifted below the two-year threshold, including those who had originally said no more, two themes emerged. First was an overall ambivalence toward pregnancy, not simply conflicted feelings but also a sense of fatalism. The majority of women in this category were in rural areas and had parity of four or more. The second theme that emerged was that the respondent had a partner who would be happy or very happy about another birth while the respondent herself did not feel strongly. For example, one 40-year old rural respondent in the Northern Region with seven children (R13.02) who changed from wanting no more children to wanting a child now indicated the decision was her own, but that she was deferential to her husband’s preference.

*I know [my husband] likes children even though we have nothing. Take a look at my house, we don’t have strength but we are managing with life… We talk all the time. He said we are going through a tough time but do we still want more children or we should try a method? And I said whatever you decide I have no problem with it… I would like to have another child any moment from now*.

In sum, 41 of 50 respondents with unmet need consistently answered the question about desirability of a/another child in the GDHS and the follow-up. The main source of inconsistency was movement from undecided to wanting another child. Including respondents who changed from wanting no more children to wanting a/another child, there were 11 cases where the stated time to next birth moved into the two-year range, seven of which (14% of unmet need sample) would have meant that women were not classified as having unmet need.

### Discrepancies in reporting of method use

At the beginning of the follow-up survey, after questions to confirm key identifying characteristics, non-pregnant women were asked if they were currently using family planning. The wording of the question was the same as in the DHS *(“Are you currently doing something or using a method to delay or avoid getting pregnant*?*”)*. Unlike in the DHS, however, women who responded “no” were prompted about natural methods *(“What about the rhythm/calendar method*? *What about withdrawal*?*”)*.

A comparison of method use reported in the GDHS and the follow-up survey is shown in Table 5. Out of 44 non-pregnant respondents that the GDHS determined to have unmet need for family planning, 15 (34 percent) reported in the follow-up interview that they actually were using a method of family planning. Five reported using a modern method, nine reported a traditional method, and one reported both. Additionally, three respondents who said they were using a method in the GDHS reported not using a method in the follow-up study.

**Table 5:** Comparison of current family planning use, as reported to the GDHS and follow-up survey.

Among non-pregnant women					
	Follow-up: Not using	Follow-up: Using traditional	Follow-up: Using modern	Total	*Discrepant*
GDHS: Not using (unmet need)	29	9	6	44	*34%*
GDHS: Using traditional method	0	4	0	4	*0%*
GDHS: Using modern method	3	3	36	42	*14%*
**Total**	**32**	**16**	**42**	**90**	*23%*

Note^:^ In the GDHS, following a section on knowledge of family planning methods, women were asked: *"Are you currently doing something or using any method to delay or avoid getting pregnant*?*”* In the follow-up survey, after identity verification, women are asked *"I’d like to begin by confirming the information I received*. *Are you currently doing something or using any method to delay or avoid getting pregnant*?*"* If the answer was no, they are asked: *"What about the rhythm method*? *What about withdrawal*?*"*

Respondents who reported discrepant use of a method in the GDHS and the follow-up survey were closely examined for identity verification questions and checked against other women in the household to see whether the follow-up interview may have been conducted with the wrong household member. None of the GDHS respondents with unmet need who reported using a method at the time of follow-up lived in a household with another woman who used a method. All matched on at least seven of nine pieces of identifying information.

Some of the women may have started or stopped a method in the intervening days between interviews. In cases where women were asked about the discrepancy, none reported starting between interviews. Two users of modern methods said that they were using a method and had told the GDHS interviewers but it was not recorded. The DHS question on method use is specifically intended to be inclusive of traditional methods *(“doing something or using a method to prevent pregnancy”*) and in the DHS interview, immediately prior to the question on current method use, respondents are asked whether they have heard of a list of methods, including rhythm or calendar, withdrawal, and lactational amenorrhea. They are read a brief description of each method type. But the main theme that emerged among respondents who reported natural methods was that they misinterpreted the GDHS question about method use as being about modern methods. For example, here is an exchange with a 26-year old woman in an urban part of Central Region (R05.06):

Interviewer: *I’d like to begin by confirming the information I received*. *Are you currently doing something or using any method to delay or avoid getting pregnant*?

Respondent: *No*, *I am not on any medication*.

Interviewer: *Yes*, *I understand you may not be on any medication to prevent pregnancy but there are other ways of preventing pregnancy such as the rhythm or withdrawal methods*. *Are you currently using any of these to prevent pregnancy*?

Respondent: *Oh*, *yes*, *I didn’t understand at first*. *I use the withdrawal method*.

Interviewer: *Please according to the information I have from the previous interviewer*, *you are currently not using any method or means to prevent pregnancy*. *Is that information correct*?

Respondent: *Yes*, *it is*. *When they came they only asked about the modern methods of family planning*. *They didn’t ask about the rhythm and the withdrawal like you did*. *That is why I said no but my husband and I use the withdrawal*.

Four other respondents reported similarly that they understood the question as being about modern methods. As a 33-year old woman in a rural part of Central Region (R04.05) stated: *“When they asked this question*, *they asked about the family planning*, *they didn’t explain that the methods included rhythm and withdrawal*, *that’s why*.*”* Overall, of the ten women who had been classified by the GDHS as having unmet need but who reported natural methods, eight initially answered “no” to a repeated GDHS question about method use and only revealed natural method use after they were asked about rhythm and withdrawal.

### Abstinence

Unmet need for family planning is designed to be computed among women who are exposed to the risk of unintended pregnancy. Exposure to the risk of pregnancy is assessed in two stages: first through self-reported sexual activity, and second through estimated fecundability. As mentioned, the definition of unmet need can apply to sexually active unmarried women as well as married women. By definition, all women who are currently in union are assumed to be at risk of pregnancy [4]. The obvious concern about this assumption is that a non-negligible proportion of women who are currently in union report long spells of sexual abstinence, for example, due to spousal migration or postpartum abstinence. A recent analysis finds that, along with fear of side effects, infrequent sexual activity is one of the two most common reasons cited for not using family planning among women with unmet need in Africa, Asia, and Latin America [7]. If all women who cited infrequent sex or abstinence as a reason for non-use were excluded from the definition of unmet need, the overall level of unmet need would decrease by a country average of 16 percent [12].

Apart from the underreporting of the rhythm and withdrawal methods, one additional type of omission that emerged from follow-up interviews was the intentional use of prolonged abstinence, particularly during the postpartum period, in order to space births. While the practice of postpartum abstinence has long been known as a dimension of birth spacing in Central and West Africa [44], and abstinence is the most effective way to prevent pregnancy, prolonged abstinence is not typically considered as a traditional method of family planning by surveys such as DHS. Respondents are asked about the date of last sexual activity separately from questions about methods to prevent pregnancy.

What is missing from DHS assessments of abstinence but emerged from the follow-up interviews is the intentionality behind abstinence. In their follow-up interview, 8 of 44 non-pregnant respondents indicated that abstinence was intentionally ongoing and was designed to prevent pregnancy. For example, one respondent, a 36-year old woman in a rural part of Northern Region (R12.05) said *“if he sleeps with me I will be pregnant*, *and I don’t want to be*, *and he is okay with the decision [to be abstinent]*.*”* Others cited lengthy durations of postpartum abstinence for the purposes of birth spacing. *“The gods we worship do not allow [family planning] so when you have a small child you avoid sex”* was how a 20-year old respondent (R10.03) described it. A 31-year old rural woman in Northern Region (R10.05) responded, *“We discussed that the child is small so we should wait for the child to grow first and because of that*, *I don’t sleep in the same room with my husband again*… *my husband said he will not touch me until my child grows*.*”*

Abstinence within marriage is typically considered a temporary state that does not preclude unmet need, as the woman may be at risk of unwanted pregnancy as soon as the abstinence ceases. Indeed, some women indicated that they were abstinent because their husbands were traveling, or that they were withholding sex because of womanizing; such abstinence is involuntary and, based on their fecundability, these respondents will be ‘in need’ of a family planning method as soon as sex resumes. But the most recurrent reason women cited for abstinence was that it was intentionally designed to avoid pregnancy, typically to allow time for their youngest child to grow, and thus unlikely to cease soon: in other words, abstinence was being used as a method of family planning.

### Abortion

The GDHS did not ask about abortion, but follow-up respondents were asked if they had ever had an abortion, and if so how many times. Underreporting of abortion is always a concern in surveys. The follow-up study prefaced the abortion question with phrasing used in prior surveys: (*“Women sometimes take steps to end their pregnancy*, *for example if they find themselves pregnant when they do not want to be*, *or when it is difficult for them to continue with their pregnancy because of opposition from their husband*, *partner*, *relatives or others*.*”)* Then, instead of asking whether women had deliberately ended a pregnancy, we asked using local terminology: *"Have you ever been in a situation where you or someone else have had to 'put your hand' to your pregnancy*?*"* If needed, interviewers clarified that they were asking about abortion. The majority of respondents reported never having had an abortion: 10 percent of women with unmet need and 28 percent of GDHS family planning users reported having had an abortion, mostly only once. The pooled result, that 19 percent of all respondents reported having ever had an abortion, is broadly consistent with the 2007 Ghana Maternal Health Survey, which found that 15 percent of all women reported having had an abortion in their lifetime [45].

### Reasons for non-use of family planning

In discussing use and non-use of family planning, five themes emerged: gaps in knowledge, fear of side effects, cost and access, the role of male partners, and religion.

#### Gaps in knowledge

Knowledge of contraception is nearly universal in Ghana; in 2014 the GDHS found that 99.5 percent of currently married women know of a method of family planning, and that women know on average nine different methods. Even so, a few interviews revealed gaps in knowledge and education. At the extreme were a small number of respondents who did not know a method or did not know how to access a method, mostly in the rural Northern part of the country. Women’s own perception of their risk of pregnancy occasionally seemed to reflect an opportunity for additional education about fecundity and biological processes.

Interviewer: *You have told me you don’t really want to have any child even though your husband wants another baby*, *please why are you not using any method to delay or prevent getting pregnant*?

Respondent (age 40, rural Central Region, R05.04): *I won’t get pregnant*. *I’ve advised my womb not to get pregnant again so I won’t get pregnant*… *I’ve learnt about family planning*. *I just don’t want to use any method*. *My womb is a family planning on its own*.

Responses that suggested a lack of knowledge about family planning or biological processes were fairly infrequent, but they do suggest that outreach to rural communities and to men, who hear about side effects mostly from rumors, may be particularly helpful. Additionally, respondents sometimes seemed to have difficulty differentiating the possible side effects of IUDs, implants, and the pill from side effects of injectables.

#### Fear of side effects

Among women classified as having unmet need, fear of side effects was by far the most commonly-cited reason for not using modern methods. The theme of side effects arose in 34 of the 50 interviews with follow-up respondents who had unmet need in the GDHS. The side effect mentioned overwhelmingly was menstrual disorders, specifically the absence of menstruation caused by hormonal methods. The second most frequent side effect mentioned was weight gain on injectables. For example, one 38-year old respondent in Greater Accra with four children (R06.05) described:

Respondent: *Previously*, *I use the injection but I stopped when I gave birth to my daughter*.

Interviewer: *So why did you stop*?

Respondent: *The side effect I had was that anytime I go in for the injection*, *I grow big and my stomach also grows big; that is why I stopped*. *With the injection*, *your menses does not come regularly*, *but as human beings our menses is supposed to come regularly*.

This quote exemplifies an important theme from discussions with respondents: the absence of menstruation was seen as not just as an undesirable trait of modern methods, but also as a marker of disease or even a possible cause of disease in the body. Other side effects, including headaches, dizziness, nausea, heart palpitations, chest pains, and high blood pressure, were mentioned infrequently. Respondents occasionally expressed that they or their partners were also concerned about difficulty conceiving after they ended use. Typically, side effects were something women themselves had experienced or had heard about from a friend or family member’s experience. In a few cases women had tried multiple modern methods without satisfaction. As one respondent explained:

*Personally, when I use those modern methods, it does not help me. It has an effect on me. After I removed the IUD, I wanted to try some method but I realized that for exactly a year it was disturbing me. For me to get to the hospital, I was helped by people to sit in a car. My waist, I could not help it at all; that is why I wanted to remove it. As I got to the hospital and told the nurse what I was going through, she said, if that is the case, for me to be free, it has to be removal. Now I am free after it was removed. After that, I went to the doing store to buy another method [pills]; when I take it, I feel a burning sensation in my whole body. I realized that none of the family planning methods are good for me, so I’ve discussed with [my husband] not to use any type of contraceptive. So at least if anything, he will withdraw. I also use my date to calculate*.

(Age 35, Greater Accra, four children, R06.10)

In addition to side effects, some respondents expressed an overall dislike of chemicals or hormones in their body, even an acceptance of some myths around modern methods. For example:

*The clinic and hospital are near us; when you step in you will get it done. The monetary aspect is not expensive, but when you consume it is expensive because it can cost you the future… I don’t want to consume more chemicals into my system… I will rather just abstain from sex*.

(Age 44, urban part of Northern Region, five children, R11.07)

Some respondents indicated that if not for their bad experience with modern methods they would have wanted years or even decades of protection against pregnancy but now are determined not to use modern methods. In some cases, their experience led them to a preference for a traditional method. For example, a 42-year old respondent in Greater Accra with three children (R05.06) described:

Respondent: *I have used the family planning before [injectable] but I developed health issues. As I speak now I have high blood pressure due to the method I used, so now I have to use the withdrawal*.

Interviewer: *So, you are currently using the withdrawal to prevent pregnancy. Why did you choose to use the withdrawal? Was it what you most preferred?*

Respondent: *Well, first of all, I want to avoid getting pregnant and secondly, I do not want to develop any other health conditions due to the side effect of a family planning method. In my case, I have a lot of issues. I do not even menstruate [on injectables]. So I think the withdrawal is better*.

Women who expressed a preference for traditional methods were often urban and educated. Although the study sample was too small to draw definitive conclusions about women of a particular educational group and area, this trend is consistent with evidence from Cameroon of a distinct preference for traditional methods among educated, urban women [46].

#### Cost and access

Follow-up respondents were asked about perceived cost and access barriers to obtaining contraception. Table 6 displays the results on cost and access barriers among non-users compared with confirmed users. Here we can see that 15 percent think modern contraceptives are difficult to access and 16 percent think they are expensive, compared to 12 and 10 percent respectively among users. The more important difference is in the don’t know category: an additional 16 percent of non-users don’t know if contraception is difficult to access, most because they have never tried to obtain it, and 38 percent don’t know if it is expensive.

**Table 6:** Comparison of perceived cost and access barriers to contraception among contraceptive users and non-users.

*Panel A*: *Among 55 follow-up respondents who said they are not using a modern method*, *percent who say*:				
	Difficult to access	Not difficult to access	Don't know	**Total**
Expensive	5	9	2	**16**
Not expensive	5	40	0	**45**
Don't know	4	20	15	**38**
**Total**	**15**	**69**	**16**	**100**
*Panel B*: *Among 41 follow-up respondents who said that they are using a modern method*, *percent who say*:				
	Difficult to access	Not difficult to access	Don't know	**Total**
Expensive	7	5	0	**12**
Not expensive	2	83	0	**85**
Don't know	0	2	0	**2**
**Total**	**10**	**90**	**0**	**100**

Note: Percentages may not sum to total due to rounding.

As indicated in Table 6, 15 percent of respondents with unmet need perceived access barriers to contraceptives. Access barriers include not only distance to health facility, waiting time, and availability of supplies, but also operating hours: a study in the Nkwanta district of Ghana found that favorable opening hours was the most significant access factor [47]. Most respondents mentioned that contraception was available in hospitals, clinics, and pharmacies. *“Every pharmacy sells some*. *Immediately you mention what you want*, *they will give it for you unless they don’t have some*,*”* said one 35-year old respondent in Greater Accra (R06.02). Another 40-year old respondent in rural Central Region (R02.07) said, *“They can easily be accessed from the hospitals and the pharmacy shops at a low cost*.*”* The only concerns about access tended to be in rural Northern villages. *“There is no way you can access the service in this village*. *We have to travel to the clinic which is far from here*, *to get the service”* (Age 37, rural Northern Region, R13.12).

Family planning is not free in Ghana, but it tends to be offered at very low cost. Among confirmed non-users of modern methods, 38 percent were unaware of cost or had had no opinion on it. Only one respondent explicitly cited a cost barrier. In general, most respondents with unmet need felt that expense did not pose a barrier. As a 41-year old woman in rural Central Region (R02.01) said, *“For something that you can use to avoid pregnancy and space your child births*, *it should have been really expensive*, *but it has been made in such a way that everyone can afford to use it*.*”* At the same time, this woman said she preferred traditional methods.

Consistent with the GDHS, in the follow-up study the majority of respondents with unmet need reported knowing a source of family planning. In general, contact with health care facilities had a positive or somewhat neutral influence on non-users. Women reported having been educated through their contact with fieldworkers and health providers. Cost and access emerged in follow-up interviews, but not as a central theme for non-use.

#### The role of husbands and partners

In discussing their decisions about family planning, interviewers asked women about the role of their husband, live-in partner, or boyfriend. Transcripts were thematically coded for whether the woman indicated at any point that her husband or partner opposed family planning and for whether he supported or accepted it. Half of the 32 confirmed non-users mentioned at least once that their husband opposed family planning. As one respondent in rural Central Region (R02.01) described, *“He always says that if the fish had decided to use family planning*, *would we have gotten any to eat*? *He is against it*.*”* However, she felt that his opinion was not important to her and she avoided family planning for other reasons. Compared with non-users, only a minority of family planning users indicated that their husband or partner opposed family planning.

In some relationships men acted as gatekeepers, either allowing or refusing family planning. One respondent, a 37-year old woman in rural Northern Region (R13.12), said *“I have to get his consent before I go in for the service*.*”* Other respondents with unmet need said that they did not have permission from their husband to use family planning and so they were not using it. Women’s empowerment within the relationship was an important factor. Some women were willing to use family planning irrespective of their husband’s opinion. For example, a 35-year old married woman in urban Central Region (R03.08) explained:

*My husband’s role is very important. You see he opposed it initially because of the rumors about side effects. So when he realized I had been using it for three years and yet had not experienced any of those side effects he had heard about, he realized it was safe after all and gave his consent. He even started encouraging and reminding me to take the pill because he realized I was only helping him and had the family’s best interest at heart*.

In other relationships, women referred to a joint discussion about family planning. One respondent who uses the pill recounted her conversation with her husband this way:

*My Mom told me that because of the way the world is today, I mean about the hardships, so we should space our births so that we don’t have to have such a hard time. So I went to tell [my husband] what she had said and he said that it doesn’t matter because when it was their turn to have children, nobody stopped them, and they had as many children as they wanted and only stopped when they were much older and couldn’t have any more. Then I told him that the economic conditions of today are not as pertained back in their days so we should try and space our births so that we will be able to cater for our children’s needs in relative comfort. So that when we’re able to, we’ll continue having other children. Then he said that even if I become pregnant now, he will be very happy. I told him that there was no way that would happen because I am using a method to prevent pregnancy. Then he said he’s okay with it*.

(Age 31, rural Central Region, four children, R04.06)

Overall, husbands and partners were both a positive and a negative influence on women’s decisions about family planning; users and non-users rated the role of their husbands or partners in their decision to use or not use a method equally.

#### The role of religion

Respondents came from a diversity of religious backgrounds, including Christian, Muslim, and atheist. In the follow-up survey women were first asked about their religion and how their religion viewed family planning. Women’s interpretation of their religion’s views on family planning varied widely. Some Christian and Muslim women said that it was opposed; others characterized their religion as being tolerant of or even encouraging family planning. Women were then asked whether their religion’s views influenced their decision to use or not use family planning.

Many women indicated that their religion was supportive of family planning or that religion was negative but did not have an influence. For example, here is one exchange with a married respondent, a 35-year old woman in a rural part of Central Region with four children (R04.07):

Interviewer: *Please can you tell me a bit about your religion’s views on family planning*.

Respondent: *They say it’s not good. According to them, the Bible says that we should be fruitful and multiply and replenish the earth so if you do that, it’s a sin against God*.

Interviewer: *So is this view the reason why you’re not using family planning? Does this view influence your decision to not use it?*

Respondent: *Oh, no. According to the Bible, God helps those who help themselves. So it may be true that that’s what the Bible says but you also have to help yourself by guarding against difficulties and hardships. So if you have to use it, you must use it. Having lots of children brings nothing but hardship*.

In Ghana, some religious practices have made an effort to promote family planning. One current IUD user who was classified as Pentecostal/Charismatic by the GDHS described the positive influence of her church:

*There was a lecture / education in our church to the women’s group on what women go through during child birth, caring for the babies, financial problems and how to space the children so that you don’t get pregnant as a Christian and cause abortion which brings problems and sin into our lives. So they have realized that family planning is good for the woman to protect herself and gives you the woman the freedom to do your work to take care of them. I went to church that day and came to inform my husband that this childbearing is something else. I told him that in order for me to not get pregnant and cause several abortions he should allow me to go and fix a family planning method. They were also featuring some advertisements about the family planning on the television, radio and everywhere you go. And he said if that is the case, if [family planning] will not bother me, then I should go for it. He even said that if we decide that we will not have any more children, then it is okay*.

(Age 42, Greater Accra, five children, R07.04)

While religious support could clearly be helpful or even conducive to initiating use of family planning, religious opposition was also an important reason for non-use. Around half of non-users said that religious opposition to family planning influenced their decision not to use family planning. These respondents tended to cite their traditional, fundamentalist Christian faith or Muslim faith as being opposed to family planning. Respondents whose religion opposed family planning tended explain in simple and absolute terms:

*My religion preaches that any means to prevent a pregnancy is equal to committing abortion and this is a sin*.

(Age 44, urban Central Region, nine children, R03.02)

*My religion says it is not good for a Muslim to use family planning*.

(Age 29, rural Northern Region, four children, R13.05)

#### Overall comparison of reasons for non-use

The GDHS survey asked most women with unmet need about their reasons for non-use in an open-ended way. *(“You have said that you do not want (a/another) child soon [or*: *You have said that you do not want any (more) children*.*] Can you tell me why you are not using a method to prevent pregnancy*? *Any other reason*?*”*) Nationwide in 2014, among married and sexually active unmarried women classified as having unmet need who were asked why they were not using a method of family planning, 49 percent stated that fear of side effects or health concerns was a reason for non-use. (Note: Multiple reasons are allowed; women who are pregnant or who have undefined fertility preferences are not asked this question.)

Toward the end of the follow-up survey, women who confirmed that they were not using family planning were asked the same open-ended question. For the 30 women who were asked the GDHS question about non-use, reinterviewed for the follow up survey, and confirmed as non-modern method users, I examined the follow-up transcript for reasons for non-use, including the repeated question when applicable. Table 7 shows the results of this comparison by reason type. Each reason and its count from the GDHS are shown. On the right-hand side, the table shows the percentage of cases where that theme was independently confirmed as a reason for non-use of family planning. I also show the number of respondents who indicated that theme as a reason for non-use in the follow-up survey but had not mentioned it in the GDHS survey. On average, respondents gave 1.1 reasons for their non-use of family planning in the GDHS but 3.0 reasons in the follow-up; the latter estimate would have been higher if respondents who reported actually using a method were excluded.

**Table 7:** Correspondence between reasons for not using family planning in the GDHS and follow-up.

Out of 30 follow-up respondents in the GDHS who were asked their reasons for not using family planning^1^ and who confirmed that they are not using a modern method, the number who independently confirmed the reason in a follow-up interview, and number of additional respondents who also had that reason						
Reason	GDHS^2^	Follow-up study				
		Independent confirmation^3^	Number confirmed	Number additional^4^	Total cases	Percent difference
Fear of side effects/health concerns or interferes with body’s processes	12	100%	12	11	**23**	+92%
Husband/partner opposed	5	60%	3	7	**10**	+100%
No sex/infrequent sex	3	67%	2	6	**8**	+167%
Cost/access/availability/source^5^	3	67%	2	0	**2**	-33%
Religious prohibition (includes opposition)^6^	2	50%	1	8	**9**	+350%
Breastfeeding/postpartum amenorrheic	3	0%	0	1	**1**	-67%
Subfecund/infecund	2	50%	1	1	**2**	+0%
Inconvenient to use	1	0%	0	0	**0**	-100%
Others opposed	1	100%	1	3	**4**	+300%
Knows no method	1	100%	1	0	**1**	+0%
**Additional reasons that emerged during follow-up**						
Respondent opposed	-	-	-	8	**8**	-
Need more information	-	-	-	2	**2**	-
Fatalistic	-	-	-	2	**2**	-
Not married	-	-	-	1	**1**	-
Plan to get soon	-	-	-	1	**1**	-
Using natural (discrepant)	-	-	-	9	**9**	-
Ambivalent fertility preferences (< 2 years)	-	-	-	7	**7**	-
Other	-	-	-	1	**1**	-
Total	**33**		**23**	**68**	**91**	
*Average number of reasons per respondent*	*1*.*1*		*0*.*8*	*2*.*3*	*3*.*0*	

^1^ Not all respondents with unmet need are asked this question. Pregnant women, women who were undecided about having another birth, and women who wanted to delay birth until after marriage, were not asked this question.

^2^ In response to the GDHS question "You have said that you do not want (a/another) child soon [or You have said that you do not want any (more) children.] Can you tell me why you are not using a method to prevent pregnancy? Any other reason?" Possible responses are not read out loud; interviewers classify each response with one of 22 pre-coded responses or "other-specify."

^3^ Percentage of the DHS users with that reason who independently gave the same reason in the follow up survey. Interviewers made no reference to the GDHS answer. Responses were considered from the entire follow-up interview and not simply from the question about non-use.

^4^ Additional respondents not identified by GDHS who explained non-use for that reason.

^5^ Includes too expensive, too far, lack of access, knows no source, preferred method not available, no method available.

^6^ The GDHS term is ‘religious prohibition’; religious opposition that the woman considered to be an important part of her decision was included on the grounds that it would have been treated similarly.

The follow-up study was interested in independently understanding unmet need; no attempt was made to quiz respondents on the validity of the reason they gave to the GDHS for non-use. The results shown in Table 7 indicate that fear of side effects, a major theme from the GDHS, was mentioned as a reason for non-use among 23 of 30 respondents. Opposition from husband or partner and religious opposition also appeared to have been underreported in the GDHS. Meanwhile, despite additional prompting on cost and access, no additional cases where cost or access posed a barrier were found.

Some respondents gave reasons not mentioned in the GDHS that are included in Table 7. Two were explicitly fatalistic about non-use of family planning (“*we are under the care of the gods so am not using anything to protect myself”* said a 20-year old respondent in the rural Northern part of Ghana, R09.06). Two said they needed more information; one planned to start family planning soon. Seven of the 30 women interviewed had revised their fertility preferences within the two-year window, and five others (R05.02, R05.04, R06.01, R09.02, R09.06) included a statement along the lines of *“I just don’t want it*,*”* or *“My heart does not like it*.*”* Reliance on abortion did not emerge as a theme for any respondents.

## Discussion

This article endeavored to describe the local meanings and lived experiences behind women’s survey responses that produce measurements of unmet need in surveys such as DHS. A complete picture of unmet need requires examining not just barriers to family planning and resistance to modern contraception but also perceptions of risk of becoming pregnant and fertility preferences that precede interest in the use of family planning. A major finding is the level of discrepancy between interviews in two key pieces of information that determine unmet need status: contraceptive use and fertility preferences. DHS respondents underreported use of family planning, particularly traditional methods. Additional prompting on traditional methods was expected to detect some underreporting of traditional method use, in line with findings from two prior surveys in West Africa that used simulated DHS questions [48, 49]. The magnitude of the omission (23 percent) was surprising, however.

An additional theme that emerged was that focusing on periodic abstinence as the only type of abstinence valid for purposes of family planning overlooks the deliberate use of abstinence in the postpartum period to avoid pregnancy. Nationwide in Ghana, nearly one-sixth of married women with unmet need reported having been abstinent since their most recent child was born.

Reproductive preferences are known to be unstable over the long term, but this study finds that for a minority of respondents fertility preferences were revised even in the span of a few weeks. Fertility preferences were also unstable among a reference group of family planning users. The most frequent change to the fertility preference questions between the GDHS and the follow-up study occurred among women who initially reported themselves as undecided on fertility preferences but shifted to wanting another child. The main type of ambivalence was in desired timing of the next birth. In 11 cases, or 25 percent of all non-pregnant women, revised fertility preferences could have affected their unmet need status.

Among women with unmet need who gave consistent information about family planning and reproductive preferences in the GDHS and the follow-up study, the most frequent reason for unmet need was fear of the side effects or health consequences of modern methods. Women frequently have had adverse reactions to hormonal methods of family planning or know someone who has. The overwhelming concern was menstrual irregularities; weight gain, headaches, dizziness, and other side effects were mentioned infrequently. In only a few cases were reports of side effects based on rumors.

In addition to fear of side effects, other reasons for not using contraception were women’s own or husband’s opposition, fatalism, and religious convictions. Earlier studies have found attitudinal resistance to family planning in Ghana and an increasing shift toward concern about side effects [33, 35]. This mixed methods study supports and extends the picture of opposition to family planning use. In follow-up discussions with respondents, opposition was more substantial than it appeared from the GDHS: on average, non-users expressed at least three reasons for not using family planning.

Ghana is a religiously diverse country. Religion had both a positive and a negative influence on women’s use of family planning. In some cases, family planning users cited religious educational outreach; other family planning users felt comfortable ignoring religious teachings that were in conflict with their own circumstances or life goals. Half of all non-users said that religion had an important influence on their decision not to use family planning.

Unmet need is measured among women, but it is important to recognize the influence of partners as well. This mixed methods follow-up study shows that the husband’s or partner’s influence appears to be equally important for users and non-users alike. Half of non-users explicitly mentioned that their husband opposed contraceptive use. Consistent with other studies, women’s empowerment appears to be an important dimension of family planning use in Ghana.

### Study limitations and strengths

#### Limitations

While the sample size interviewed is quite large for a qualitative study, it should not be considered regionally representative. Clusters were selected from among those available for follow-up during the study time period, with some desire for geographic diversity and balance between urban and rural areas. Study clusters overrepresent rural and poorer areas; ambivalent fertility preferences and underreporting of traditional methods may thus be more common among respondents than among the regional population.

#### Strengths

Embedding a mixed methods study design within the GDHS survey proved advantageous. First, the follow-up study benefited from the rigorous and standardized sampling and household listing process undertaken by DHS surveys. By definition, the main group of interest for this study was married or sexually active, fecund women who want no more children but who are not using family planning. These women are particularly difficult to locate through facility-based convenience sampling. Current users of family planning in the same clusters could be included as a reference group. Second, the study was aided by a rich array of information already gathered about respondents’ demographic characteristics, reproductive histories, and familial context in the GDHS. Access to GDHS data meant that interviews could be conducted more quickly than otherwise possible; moreover, a small number of identical follow-up questions could provide insight on the quality and consistency of extant data. Third, mixed methods approaches are valuable as they help overcome weaknesses inherent in either a purely quantitative or qualitative approach [50, 51]. By including a mixture of closed- and open-ended questions the study was able to help answer key questions about unmet need in Ghana.

## Conclusion

The study findings have several major implications. First, although the prevalence of unwanted pregnancies attests to an ongoing need for family planning, Ghanaian women appear to manage their reproductive lives with more agency than may be apparent from survey data. Almost one-third of the respondents identified as having unmet need were either using a traditional method or intentionally abstinent as a method of child spacing. These women would still be defined as having an unmet need for modern contraception, but as previous studies from West Africa have found, women may be knowledgeable about modern methods but prefer traditional methods for a number of reasons [46]. Unobserved use of traditional and natural methods helps shed light on how Ghanaian women maintain fairly low fertility in regional perspective despite low reported levels of family planning use.

Second, even if we focus only on women with an unmet need for modern methods, the group defined as having an unmet need appears to be somewhat unstable, even in the very short-term. Some women, particularly in the rural Northern part of the country, expressed ambivalence and changing preferences about timing of the next birth even within a week of their original interview. This finding speaks to the importance of recognizing and accounting for women with unstable fertility preferences when designing programmatic interventions.

Third, this study underscores the possibility that opposition to modern methods among non-users is frequently more substantial than what is apparent from survey data. Women who are interested in modern methods but not using them because of lack of knowledge, cost, or access are relatively scarce, even in isolated, rural communities. This is already known from survey data; what this study adds is that opposition to modern methods may be more substantial than generally believed. Many non-users studied cited multiple reasons for non-use: fear of side effects, partner opposition, personal opposition, and religious opposition. Thus while increasing method choice and ensuring safe access to family planning are critically important, the results strongly suggest that additional demand generation and outreach activities appear to be necessary prerequisite to sustained increases in modern contraceptive use.

A previous study found that the most intractable reasons for not using family planning are attitudinal and religious resistance; in contrast, concerns about side effects are more easily overcome with education and information [52]. The present study confirms that women with attitudinal resistance frequently have multiple layers of resistance to modern methods (religious, partner, personal). Additionally, there appears to be strong opposition to modern methods among women who have themselves experienced negative side effects. Certainly there appear to be opportunities for education about reproductive biology or outreach to dispel myths about modern methods. But ambitious efforts to scale up use of modern methods should continue to guard against any risk of becoming unintentionally coercive at the local level.

Ghana has a relatively strong family planning program. Notably, the reference group of family planning users expressed satisfaction with their methods. There are opportunities for improvement, of course. Ready and safe access to methods should be routine. High-quality care should be standard. The method mix could be expanded: a few non-users expressed a desire for a longer-acting method or even sterilization. Programs providing client-centered or couple-centered counseling about contraception that supports total method choice and discussion about side effects from modern methods should be supported and expanded.

The evidence presented here suggests additional attention should be paid to measurement of traditional and natural methods in contemporary large-scale surveys. To the extent that traditional methods are undercounted, some natural methods considered modern, such as standard days method, lactational amenorrhea, and cycle beads, may also be undercounted as well. Additionally, while current programmatic efforts focus on modern methods, it is still important to understand what proportion of users of non-modern methods already consider themselves protected from the risk of pregnancy. Complete, prolonged abstinence is the most effective way to avoid pregnancy, yet the literature on traditional family planning methods is notably silent about complete abstinence, recognizing only periodic abstinence. This produces a kind of tautology: abstinence as a method of family planning is not measured because it is not recognized, and it remains invisible because its intentional use is not well-measured by contemporary surveys. The present study suggests that some consideration be given to prolonged abstinence as a method of family planning when it is intended to limit or space births.

Apart from complete abstinence, modern contraceptive methods are the most effective form of family planning. They also offer women the opportunity to initiate birth spacing and limitation independently of their male partners and even surreptitiously, thus giving women who may have little voice within their marriage the power to shape their own reproductive lives. Efforts to empower women within marriage would likely have a beneficial spillover effect on the use of family planning; moreover, given the strong influence of male partners, outreach to men should be an important part of efforts to scale-up family planning.

At a macro level, long-acting reversible contraceptive methods are an important source of long-term protection, but women frequently discontinue implants and injectables due to side effects and method-related reasons [53]. While the absence of menstruation may be seen as an indirect benefit of contraception in some cultures and contexts, non-users in Ghana perceive the absence of menstruation as particularly problematic; in some cases as a sign of disease or a cause of health problems. In the long-term, expanding access to the non-hormonal IUD in Ghana could help meet the needs of women who are opposed to methods that affect their menstrual cycles and who want long-term protection. Access to the IUD in Ghana is low [54].

A few women also indicated that they would prefer a permanent method. Sterilization is not without controversy and concerns over coercion, but it is a non-hormonal method that could be offered as part of total method choice in Ghana. Additionally, clinical development of contraceptive methods that have fewer side effects—particularly methods that do not affect menstruation—could also be an important strategy to address the unmet contraceptive needs of women and couples in Ghana and other developing countries.

## Supporting information

S1 File Study questionnaires. (PDF)
