# Supplementary material for: Fear, opposition, ambivalence, and omission: Results from a follow-up study on unmet need for family planning in Ghana
Source: PLoS One. 2017 Jul 31;12(7):e0182076. doi: 10.1371/journal.pone.0182076 (PMC5536298; doi:10.1371/journal.pone.0182076)

Questionnaires for Follow-Up Unmet Need Study in Ghana

This is a paper rendering of questionnaires that were implemented on Android tablets using Mobile Data Studio software.

Text in blue indicates instructions to the interviewer. Text in **bold** may be read to respondent, if applicable. Text in green indicates DHS data entry that should display on the tablet.

After this introductory section, based on responses to DHS, a computer program directs to the questionnaire for the respondent's group:

Group A: non-pregnant women with unmet need
Group B: pregnant women with unmet need
Group C: current users of any method except sterilization

Identification

- use structure number, map, and name of head of household to identify location
- inquire about woman based on relationship to head of HH and her year of birth
- politely ask if she is available to be interviewed, and if not to recommend a time to return

Verbal consent

Hello, my name is _________. I am working with ISSER/University of Ghana. We are conducting a study in order to find better ways to help couples achieve their family goals. During the recent interview with my colleagues, you stated that you would be willing to have someone from our team follow-up with you. If you still agree to participate, I will ask you questions about pregnancy, having children, and family planning. The questions usually take about 20 to 30 minutes. All of the answers you give will be confidential and will not be shared with anyone other than members of our survey team. You don't have to be in the survey, but we hope you will agree to answer the questions since your views are important. If I ask you any question you don't want to answer, just let me know and I will go on to the next question or you can stop at any time. We would like to record the conversation to help us remember what you said.

GIVE CARD WITH CONTACT INFORMATION

Do you have any questions for me?

May I have your permission to interview you today? YES NO

May I have your permission to record the interview? YES NO

Signature of interviewer ______________________________ Date, Time ________________

Prior to interview

- Speak identifying information into the microphone: date, cluster number, household number, woman's line number, your name
- Position audio recorder near the interviewee, with one microphone toward you and one toward her.

- Turn audio recorder on; look for red light

<u>Verification</u>

Just to be certain I am speaking with the correct person, can you please tell me your year of birth? SELECT FROM 1969 – 2000 Display DHS year. Ask about any discrepancies: From the information I received, I understood that your year of birth was ____.
And can you please tell me your month of birth? SELECT FROM 1-12 OR 98 FOR DK Display DHS month. Ask about any discrepancies with DHS month of birth
Are you currently married or living with a man as if married? YES-MARRIED / YES-LIVING WITH A MAN / NO-NOT IN UNION Display DHS marital status. Ask about any discrepancies.
Have you ever given birth? YES NO Display DHS answer. Ask about any discrepancies.
If YES: **How many sons do you have living with you now**? **How many daughters do you have living with you now?** Display DHS answer. Ask about any discrepancies.

If several pieces of this information do not match DHS, double check relationship to household head, household number, inquire further before terminating interview.

Group A: not pregnant, fecund, not using any method and who want birth at least 2 years away or not at all

Remember: turn audio recording on. Speak identifying information into the microphone: date, cluster number, household number, woman's line number, your name

I would like to begin by just confirming the information I received. Are you currently doing something or using a method to delay or avoid getting pregnant? YES NO
Are you using the rhythm method? YES NO
Are you using withdrawal? YES NO
If YES TO ANY OF ABOVE: From the information I received I understood that you were not using a method to delay or avoid getting pregnant. Did I receive incorrect information?
(Politely ask her to explain)
INTERVIEWER – STOP AND move to Group C

1 **At this point in your life, what do you think would be some positive aspects about becoming pregnant and having (a/another) child?**
What would be some of the negative aspects about becoming pregnant and having (a/another) child?
Probe on issues like cost, health, joy, esteem.
What value, if any, do you feel (a/another child) would bring to your household?
How did you come to feel this way?

2 **How happy or unhappy would you be if you became pregnant in the next few months?** *(wait for a response)*
Please tell me more about that. What are some of the reasons you feel this way?
[SHOW RESPONDENT THE PRINTED SCALE FROM 0 TO 5]
Using a scale of 0 to 5, where 0 indicates very unhappy and 5 indicates very happy, how happy or unhappy would you be? 0 1 2 3 4 5

3 **What about your (partner/husband), how happy or unhappy do you think he would be if you became pregnant in the next few months?**
(wait for a response)

Using a scale of 0 to 5, where 0 indicates very unhappy and 5 indicates very happy, how happy or unhappy do you think he would be? 0 1 2 3 4 5
Please tell me more about that. What are some of the reasons you think he feels that way?
Is it something you have ever discussed? YES NO

- If YES: **please tell me about the last time you talked about this; what did he say…what did you say?**
- If NO: **what do you think would happen if you tried to discuss this issue?**

4 **Now I would like to understand more about your current situation.**
How likely do you think it is that you will become pregnant in the next few months?

Using a scale of 0 to 5, where 0 indicates no chance and 5 indicates high chances, how likely do you think it is that you will become pregnant in the next few months?
0 1 2 3 4 5

- ➔ *If 0, proceed to 4a/4b/4c. OTHERWISE, PROCEED TO QUESTION 5.*

4a **Why is that?**
For women who indicate 0 because their husband or partner is away: **When do you expect your (husband/partner) to return?**
Do you expect to be using a method when he returns?
How often does your partner migrate (does he have another job somewhere?)

4b *For women who indicate 0 because they are not sexually active:* **When was the last time you had sex?**
Do you expect to resume being sexually active in the near future?

4c *For women who indicate 0 due to infecundity:* **How many months ago was your last menstrual period**?

5 **Now I have some questions about the future. Would you like to have (a/another) child, or would you prefer not to have any (more) children?**
[Compare her response to DHS Q704 - ask about any discrepancies]
HAVE A/ANOTHER CHILD (go to 5A) NO MORE (go to 5B) DON'T KNOW/UNDECIDED (go to 5C)

5a If Q5 is A/ANOTHER: **How long would you like to wait from now before the birth of (a/another) child? __________________** (encourage respondent to give a range, if appropriate)
[DHS Q705a - ask about any discrepancies, factoring in time between interviews]
Please tell me more about that. What are some of the reasons you feel this way?
(probe for contingencies, for example related to income, partnership, etc.).

Now, please tell me how strongly you feel about waiting that long to get pregnant. Please give me a number between 0 and 5, where 0 means you don't mind becoming pregnant before the time you stated and 5 means you really want to avoid getting pregnant before that time.
0 1 2 3 4 5

Did you come to this decision alone or through discussing with others? ALONE / OTHERS

- ➢ If WITH OTHERS- **can you tell me about the discussions that led to this decision**
- ➔ *PROCEED TO QUESTION 7*

5b If Q5 is NO MORE CHILDREN: **Tell me about your decision to not have any more children. What are some of the reasons you feel this way?**

Did you come to this decision alone or through discussing with others? ALONE / OTHERS

- If WITH OTHERS- **can you tell me about the discussions that led to this decision**

Now, please tell me how strongly you feel about not having any more children. Please give me a number between 0 and 5, where 0 means you don't mind having a/another child and 5 means you really want to avoid having a/another child
0 1 2 3 4 5

Have you always had an intention to stop after (current number of children), or was it a recent decision?

➔ *PROCEED TO QUESTION 7*

5c If Q5 is I DON'T KNOW: **I heard you say that you don't know if you want another child or not. Could you please elaborate on that for me?**

Interviewer – can also ask her if she needs more time to think about it.

6 SKIP

7 **Have you ever tried to avoid sex with your (husband/partner) in order to prevent getting pregnant?** YES NO

- If YES- **How often do you do this?** [how often have you done this in the past?]

8 **Have you ever used anything or tried in any way to delay or avoid getting pregnant?** [compare with DHS Q313]
YES (go to 8a) NO

8a If Q8 is YES: **What methods have you used?** SELECT FROM METHOD LIST [compare with DHS Q314]
For first method: **Tell me about the reasons why you chose that method.**
[wait for a response].
What was your experience with that method? Why did you stop?
REPEAT FOR EACH ADDITIONAL METHOD USED IN THE PAST 5 YEARS.

9 **Have you and your partner ever discussed using a method to delay or avoid pregnancy?** YES NO

- If NO--**How do you think he feels about it? What does it mean to you to not discuss?**
- If YES--**Please share with me what you discussed.** (in particular, whose idea was the method, was there opposition, discussion of side effects, choice, costs, etc.)

10 **Women sometimes take steps to end their pregnancy, for example if they find themselves pregnant when they do not want to be, or when it is difficult for them to continue with their pregnancy because of opposition from their husband,**

partner, relatives or others. Have you ever been in a situation where you or someone else have had to "put your hand" to a pregnancy [induce an abortion]**?**
YES NO

- If YES: **how long ago was the most recent situation?**
  What did you do to end this pregnancy?
  Where did you seek assistance?
  What is the main reason you decided to end this pregnancy?
  Was this the first time or has it happened before? FIRST TIME / HAPPENED BEFORE
- If BEFORE: **how many times have you ended a pregnancy?**

11 *Look at Q5:* **You have said that you do not want (a/another) child soon / OR you have said that you do not want any (more) children. Can you tell me why you are not using a method to prevent pregnancy?**
Please tell me more about that. Any other reason? *(probe for detail of opposition)*

11a *If the woman cited a fear of side effects:* **What kind of side effects are you concerned about?**
How did you come to believe that it causes side effects?

11b *If the woman cited her or her own or her husband's opposition to using contraception, probe answer*: **Tell me more about how you and/or your husband came to believe that contraception was not a good choice for your family.**

12 **Now I would like to ask you about some other issues related to childbearing and family planning.**

12a **Do you think that contraception is expensive?** YES NO DK/A BIT
Please tell me more about that.
Do you think that it is difficult to access contraception? YES NO DK/A BIT
Please tell me more about that.

12b **Can you please tell me a little bit about your religion's views on family planning? Do these views influence your decision to (use/not use/have used/not have used) family planning?** YES NO DK/A LITTLE

What about the role of your husband or partner, if any, on your decision to (use/not use/have used/not have used) a method to prevent pregnancy. How important an influence is he on your decision about family planning?
IMPORTANT SOMEWHAT IMPORTANT NEITHER SOMEWHAT UNIMPORTANT UNIMPORTANT DON'T KNOW
Please tell me more about that.

What about the role of other family members? How important of an influence are they on your decision about family planning?
IMPORTANT SOMEWHAT IMPORTANT NEITHER SOMEWHAT UNIMPORTANT UNIMPORTANT DON'T KNOW

Please tell me more about that. [which other family members - siblings, in-laws, parents?]

13 **Now I would like to ask you some questions about your contact with health workers, medical staff, and health facilities.**

13a **In the last 12 months, were you visited by a fieldworker who talked to you about family planning?** YES NO [ask about any discrepancies with DHS Q326]

- If YES - **please tell me about those conversations - what did they say / what did you say?**
  What role did this information or discussion have in your decision to not use a method?

13b **In the last 12 months, have you visited a health facility for care for yourself or your children?** YES NO
[ask about any discrepancies with DHS Q327]

- If YES: **Did any staff member at the health facility speak to you about family planning methods?** YES NO
  [ask about any discrepancies with DHS Q328] **What did they say / what did you say?**

13c [IF HAS EVER GIVEN BIRTH] **Have you given birth at a health facility in the past 5 years?** YES NO [no: go to 13d]

- If YES: **Can you tell me about the last delivery you had at a health facility. At that time, did anyone come to you to discuss options for spacing births or preventing another pregnancy?** YES NO
- If YES – **tell me about those conversations.**
- ➔ PROCEED TO QUESTION 14

13d If NO to 13c: **Did anyone attend to you at your most recent birth?** YES NO

- If YES: **Who attended to you**? [type of provider]
  Interviewer - if it was a medical provider, including TBA: **At that time, did the provider approach you to discuss options for spacing births or preventing another pregnancy?** *[tell me about those conversations.]*

14 **I'm interested in learning more about your community. Are you aware of which methods, if any, the women in your community use? Please describe.**
What kinds of women do you think use these methods?
if respondent did not mention natural methods:
What kinds of women do you think use natural methods like withdrawal and the rhythm method to prevent pregnancy?

15 **Do you feel that family planning services are equally available to married and unmarried women in your community?** YES NO DK
Why/why not?

16 **Now, thinking about the future: do you think you will use a method to delay or avoid pregnancy at any time in the future?** [compare with DHS Q711]
YES [go to 16a] NO [go to 16b]

16a If YES to Q16: **What method(s) are you considering?**
Can you tell me some of your reasons for considering that method/those methods.
How will you know when the time is right?
If considering modern methods: **Do you know how you would obtain that method?**
➔ Proceed to Q17

16b If NO to Q16: **How certain are you that you will not use a method?**
Can you tell me some of your reasons.
Are there other factors that would influence your decision, such as your partner's preferences, cost, or accessibility?

17 **I have asked you a lot of questions today about childbearing and pregnancy prevention. Do you have additional thoughts on these matters that you would like to share with me?**

Thank you for taking the time to talk with me today.

Group B: pregnant women who did not want current pregnancy at all or wanted it later

Remember: turn audio recording on. Speak identifying information into the microphone: date, cluster number, household number, woman's line number, your name

I would like to begin by just confirming the information I received. Are you pregnant now? YES NO

[if YES, proceed.

If NO, ask about discrepancy with previous survey.

Then ask if she is currently using a method to prevent pregnancy. If yes, move to Group C, If no, move to group A.]

1 **At this point in your life, what do you think are some positive aspects about being pregnant and having (a/another) child?**

What are some of the negative aspects about being pregnant and having (a/another) child? *Probe on issues like cost, health, joy, esteem.*

What value, if any, do you feel a/another child will bring to your household?

How did you come to feel this way?

2 **When you got pregnant, did you want to get pregnant at that time?** [Compare with DHS Q228 - ask more if she says yes, was something she had been thinking about for a while?] YES NO

- If NO: **did you want to have a baby later on or did you not want any more children?** LATER NO MORE [DHS Q229 - ask about discrepancies]

How happy or unhappy are you that you are pregnant? (wait for a response)

Please tell me more about that. What are some of the reasons you feel this way? (wait for a response)

[SHOW RESPONDENT THE SCALE FROM 0 TO 5]

Using a scale of 0 to 5, where 0 indicates very unhappy and 5 indicates very happy, how happy or unhappy are you?

0 1 2 3 4 5

3 **What about your partner/husband, how happy or unhappy do you think he is that you are pregnant?** *(wait for a response)*

Using a scale of 0 to 5, where 0 indicates very unhappy and 5 indicates very happy, how happy or unhappy do you think he is?

0 1 2 3 4 5

Please tell me more about that. What are some of the reasons you think he feels that way?

Is it something you have ever discussed? YES NO

- If YES: **please tell me about the last time you talked about this; what did he say/what did you say?**
- If NO: **what do you think would happen if you tried to discuss this issue?**

4 SKIP

4a SKIP

4b SKIP
4c SKIP

5 *Re-ask DHS question:* **Now I have some questions about the future. After the birth of the child you are expecting now, would you like to have (a/another) child, or would you prefer not to have any more children?** [DHS Q703 - ask about any discrepancies, factoring in time between interviews]
HAVE A/ANOTHER CHILD (go to 5A) NO MORE (go to 5B) DON'T KNOW/UNDECIDED (go to 5C)

5a If Q5 is A/ANOTHER: **After the birth of the child you are expecting now, how long would you like to wait from now before the birth of another child?**
________________ (encourage respondent to give a range, if appropriate)
[DHS Q705b - ask about any discrepancies]
Please tell me more about that. What are some of the reasons you feel this way?
(probe for contingencies, for example related to income, partnership, etc.).

Now, please tell me how strongly you feel about waiting that long after the birth of the child you are expecting now to get pregnant again. Please give me a number between 0 and 5, where 0 means you don't mind becoming pregnant again before then and 5 means you <u>really want to</u> avoid getting pregnant again before then.
0 1 2 3 4 5
Did you come to this decision alone or through discussing with others? ALONE OTHERS

- If WITH OTHERS- **can you tell me about the discussions that led to this decision**
- ➔ PROCEED TO QUESTION 7

5b If Q5 is NO MORE: **Tell me about your decision to not have any more children after the child you are expecting now.**
What are some of the reasons you feel this way?
Did you come to this decision alone or through discussing with others? ALONE WITH OTHERS

- If WITH OTHERS- **can you tell me about the discussions that led to this decision**

Now, please tell me how strongly you feel about not having any more children after the child you are expecting now. Please give me a number between 0 and 5, where 0 means you don't mind having a/another child and 5 means you <u>really want to</u> avoid having a/another child after the child you are expecting now
0 1 2 3 4 5

Have you always had an intention to stop after __ children, or was it a recent decision?

- ➔ PROCEED TO QUESTION 7

5c If Q5 is I DON'T KNOW: **I heard you say that you don't know if you want another child or not. Could you please elaborate on that for me?**

Interviewer – can also ask her if she needs more time to think about it.

6 **SKIP**

7 **Have you ever tried to avoid sex with your husband/partner in order to prevent getting pregnant?** YES NO

- If YES- **How often have you done this?** [how often have you done this in the past?]

8 **Have you ever used anything or tried in any way to delay or avoid getting pregnant?** [compare with DHS Q313] YES (go to 8A) NO (go to Question 9)

8a If Q8 is YES: **What methods have you used?** [compare with DHS Q314]

For first method: **Tell me about the reasons why you chose that method.** [wait for a response].

What was your experience with that method? Why did you stop?

REPEAT FOR EACH ADDITIONAL METHOD USED IN THE PAST 5 YEARS.

Were you using any of these methods to avoid pregnancy prior to becoming pregnant? YES NO

- If YES: **what happened that you became pregnant** (for example does she think she forgot to take a pill, or does she think the method failed her)?

9 **Prior to your current pregnancy, had you and your partner ever discussed using a method to delay or avoid pregnancy**? YES NO

- If YES--**Please share with me what you discussed.** (in particular, whose idea was the method, was there opposition, discussion of side effects, choice, costs, etc.)
- If NO-- **How do you think he feels about it? What does it mean to you to not discuss?**

What about your current pregnancy, have you and your partner discussed using a method to delay or avoid another pregnancy after your baby is born?

10 **Women sometimes take steps to end their pregnancy, for example if they find themselves pregnant when they do not want to be, or when it is difficult for them to continue with their pregnancy because of opposition from their husband, partner, relatives or others. Have you ever been in a situation where you or someone else have had to "put your hand" to a pregnancy** [induce an abortion]?

YES (continue) NO (go to 12)

- If YES: **how long ago was the most recent situation? What did you do to end this pregnancy?**
  What is the main reason you decided to end this pregnancy?
  Where did you seek assistance?
  Was this the first time or has it happened before?
- If BEFORE: **how many times have you ended a pregnancy?**

11 SKIP

12 **Now I would like to ask you about some other issues related to childbearing and family planning.**

12a **Do you think that contraception is expensive?** YES NO DK/A LITTLE
Please tell me more about that.
Do you think that it is difficult to access contraception? YES NO DK/A LITTLE
Please tell me more about that.

12b **Can you please tell me a little bit about your religion's views on family planning? Do these views influence your decision to (use/not use/have used/not have used) family planning?** YES NO DK/A LITTLE

What about the role of your husband or partner, if any, on your decision to (use/not use/ have used/ not have used) a method to prevent pregnancy? How important of an influence is he on your decision about family planning?
IMPORTANT SOMEWHAT IMPORTANT NEITHER SOMEWHAT UNIMPORTANT UNIMPORTANT DON'T KNOW
Please tell me more about that.

What about the role of other family members? How important of an influence are they on your decision about family planning?
IMPORTANT SOMEWHAT IMPORTANT NEITHER SOMEWHAT UNIMPORTANT UNIMPORTANT DON'T KNOW
Please tell me morePlease tell me more about that. [which other family members - siblings, in-laws, parents?]

13 **Now I would like to ask you some questions about your contact with health workers, medical staff, and health facilities.**

13a **In the last 12 months, were you visited by a fieldworker who talked to you about family planning?** YES NO [ask about any discrepancies with DHS Q326]
If YES - **please tell me about those conversations - what did they say / what did you say?**
What role did this information or discussion have in your decision to use a method?

13b **In the last 12 months, have you visited a health facility for care for yourself or your children?** YES NO [ask about any discrepancies with DHS Q327]

- If YES- **Did any staff member at the health facility speak to you about family planning methods?** [ask about any discrepancies with DHS Q328] **What did they say / what did you say?**

13c [IF HAS EVER GIVEN BIRTH] **Have you given birth at a health facility in the past 5 years?** YES (proceed) NO (go to 13d)

- If YES: **Can you tell me about the last delivery you had at a health facility. At that time, did anyone come to you to discuss options for spacing births or preventing another pregnancy?** YES NO

If YES – **tell me about those conversations.** PROCEED TO QUESTION 15

13d If NO to 13c: **Did anyone attend to you at your most recent birth?** YES NO
If YES: **Who attended to you?**
Interviewer - if it was a medical provider, including TBA: **At that time, did the provider approach you to discuss options for spacing births or preventing another pregnancy?** *[tell me about those conversations.]*

14 **I'm interested in learning more about your community. Are you aware of which methods, if any, the women in your community use? Please describe. What kinds of women do you think use these methods?**
if respondent did not mention natural methods: **What kinds of women do you think use natural methods like withdrawal and the rhythm method to prevent pregnancy?**

15 **Do you feel that family planning services are equally available to married and unmarried women in your community?**
YES NO DK
Why/why not?

16 **Now, thinking about the future, do you intend to breastfeed your baby? Tell me about that** (ask specifically about duration and exclusivity).
After the child you are expecting now, do you think you will use a method to delay or avoid pregnancy at any time in the future?
YES (go to 16a) NO (go to 16b)

16a If YES to Q16: **What method(s) are you considering?**
Can you tell me some of your reasons for considering that method/those methods.
How will you know when the time is right?
If modern methods: **Do you know how you would obtain that method/those methods?**

16b If NO to Q16: **How certain are you that you will not use a method?**
Can you tell me some of your reasons.
Are there other factors that would influence your decision, such as your partner's preferences, cost, or accessibility?

17 **I have asked you a lot of questions today about childbearing and pregnancy prevention. Do you have additional thoughts on these matters that you would like to share with me?**
Thank you for taking the time to talk with me today.

Group C: current users of any method except sterilization

Remember: turn audio recording on. Speak identifying information into the microphone: date, cluster number, household number, woman's line number, your name

I would like to begin by just confirming the information I received. Are you currently doing something or using a method to delay or avoid getting pregnant, including rhythm or withdrawal?

If YES: **please tell me what method(s) you are using**. Record as many as possible from the following list: FEMALE STERILIZATION, MALE STERILIZATION, IUD, INJECTABLES, IMPLANTS, PILL, CONDOM, FEMALE CONDOM, DIAPHRAGM, FOAM/JELLY, LACTATIONAL AMEN. METHOD, RHYTHM METHOD, WITHDRAWAL, OTHER MODERN METHOD, OTHER TRADITIONAL METHOD.

If NO: From the information I received I understood that you were not using a method to delay or avoid getting pregnant. Did I receive incorrect information?

(Politely ask her to explain)

INTERVIEWER – STOP and move to Group A Questionnaire

1 **At this point in your life, what do you think could be some positive aspects about becoming pregnant and having (a/another) child?**

What would be some of the negative aspects about becoming pregnant and having (a/another) child? *Probe on issues like cost, health, joy, esteem.*

What value, if any, do you feel a/another child would bring to your household?

How did you come to feel this way?

2 **How happy or unhappy would you be if you became pregnant in the next few months?** *(wait for a response)*

Please tell me more about that. What are some of the reasons you feel this way?

(wait for a response)

[SHOW RESPONDENT THE PRINTED SCALE FROM 0 TO 5]

Using a scale of 0 to 5, where 0 indicates very unhappy and 5 indicates very happy, how happy or unhappy would you be?

0 1 2 3 4 5

3 **What about your partner/husband, how happy or unhappy do you think he would be if you became pregnant in the next few months?** *(wait for a response)*

Using a scale of 0 to 5, where 0 indicates very unhappy and 5 indicates very happy, how happy or unhappy do you think he would be?

0 1 2 3 4 5

Please tell me more about that. What are some of the reasons you think he feels that way? Is it something you have ever discussed? YES NO

- If YES: **please tell me about the last time you talked about this; what did he say/what did you say?**
- If NO: **what do you think would happen if you tried to discuss this issue?**

4 **Now I would like to understand more about your current situation. How likely do you think it is that you will become pregnant in the next few months?** *(wait for a response)*
Using a scale of 0 to 5, where 0 indicates no chance and 5 indicates high chances, how likely do you think it is that you will become pregnant in the next few months?
0 1 2 3 4 5

4a SKIP
4b SKIP
4c SKIP

5 **Now I have some questions about the future. Would you like to have (a/another) child, or would you prefer not to have any (more) children?**
[Compare with DHS Q704 - ask about any discrepancies]
HAVE A/ANOTHER CHILD (go to 5a) NO MORE (go to 5b) DON'T KNOW/UNDECIDED (go to 5c)

5a If Q5 is A/ANOTHER: **How long would you like to wait from now before the birth of (a/another) child? __________________** (encourage respondent to give a range, if appropriate)
[Compare with DHS Q705a - ask about any discrepancies]
Please tell me more about that. What are some of the reasons you feel this way?
(probe for contingencies, for example related to income, partnership, etc.).
Please give me a number between 0 and 5, where 0 means you don't mind becoming pregnant before the time you stated and 5 means you <u>really want to</u> avoid getting pregnant before that time.
0 1 2 3 4 5
Did you come to this decision alone or through discussing with others? ALONE OTHERS

- If WITH OTHERS- **can you tell me about the discussions that led to this decision**
- ➔ PROCEED TO QUESTION 6

5b If Q5 is NO MORE: **Tell me about your decision to not have any more children. What are some of the reasons you feel this way?**
Did you come to this decision alone or through discussing with others? ALONE OTHERS

Now, please tell me how strongly you feel about not having any more children. Please give me a number between 0 and 5, where 0 means you don't mind having a/another child and 5 means you <u>really want to</u> avoid having a/another child
0 1 2 3 4 5

Have you always had an intention to stop after ___ children, or was it a recent decision?

➔ PROCEED TO QUESTION 6

5c If Q5 is I DON'T KNOW: **I heard you say that you don't know if you want another child or not. Could you please elaborate on that for me?**
Interviewer – can also ask her if she needs more time to think about it.

6 **You said you are using a method to delay or avoid getting pregnant. Are your current methods the ones you most wanted to use?** YES NO

7 **Have you ever tried to avoid sex with your husband/partner in order to prevent getting pregnant?** YES NO
- If YES- **How often have you done this?**

8 **Prior to your current method, have you ever used anything or tried in any way to delay or avoid getting pregnant?** YES NO
- If YES: **What other methods have you used to prevent pregnancy?**

8a **Now, I understand that you are currently using a method to prevent pregnancy… tell me about the reasons you chose this method.** [wait for a response].

9 **Do you think your partner is aware that you are using your current methods?** (YES/NO)?
Have you and he ever discussed using (NAME(S) OF CURRENT METHOD(S)) **to avoid pregnancy?** YES NO
- If YES--**Please tell me about those discussions**.
- If NO-- **How do you think he feels about it? What does it mean to you to not discuss?**

Have you discussed your current method with anyone besides your partner, such as a friend or relative? YES NO
- If YES--**please tell me who / please tell me about those discussions.**

10 **Women sometimes take steps to end their pregnancy, for example if they find themselves pregnant when they do not want to be, or when it is difficult for them to continue with their pregnancy because of opposition from their husband, partner, relatives or others. Have you ever been in a situation where you or someone else have had to "put your hand" to a pregnancy** [induce an abortion]?
YES NO
- If NO: *proceed to Question 12*
- If YES: **how long ago was the most recent situation?**
  What did you do to end this pregnancy?
  Where did you seek assistance?
  What is the main reason you decided to end this pregnancy?
  Was this the first time or has it happened before?
- If BEFORE: **how many times have you ended a pregnancy?**

11 SKIP
11a SKIP

11b SKIP

12 **Now I would like to ask you about some other issues related to childbearing and family planning.**

12a **Do you think that contraception is expensive?** YES NO DK/A BIT
Please tell me more about that.
Do you think that contraception is difficult to access? YES NO DK/A BIT
Please tell me more about that.

12b **Can you please tell me a little bit about your religion's views on family planning? Do these views influence your decision to (use/not use/have used/not have used) family planning?** YES NO DK/A BIT
What about the role of your husband or partner, if any, on your decision to (use/not use/have used/not have used) a method to prevent pregnancy. How important of an influence is he on your decision about family planning?
IMPORTANT SOMEWHAT IMPORTANT NEITHER SOMEWHAT UNIMPORTANT UNIMPORTANT DON'T KNOW
Please tell me more about that.
What about the role of other family members? How important of an influence are they on your decision about family planning?
IMPORTANT SOMEWHAT IMPORTANT NEITHER SOMEWHAT UNIMPORTANT UNIMPORTANT DON'T KNOW
Please tell me more about that. [which other family members – siblings, in-laws, siblings?]

13 **Now I would like to ask you some questions about your contact with health workers, medical staff, and health facilities.**

13a **In the last 12 months, were you visited by a fieldworker who talked to you about family planning?** YES NO [ask about any discrepancies with DHS Q326]
- If YES - **please tell me about those conversations - what did they say / what did you say?**
  What role did this information or discussion have in your decision to use a method?

13b **In the last 12 months, have you visited a health facility for care for yourself or your children?** YES NO [ask about any discrepancies with DHS Q327]
- If YES- **Did any staff member at the health facility speak to you about family planning methods?** [ask about any discrepancies with DHS Q328]
  What did they say / what did you say?

13c [IF HAS EVER GIVEN BIRTH] **Have you given birth at a health facility in the past 5 years?** YES (proceed) NO (go to 13d)
- If YES: **Can you tell me about the last delivery you had at a health facility. At that time, did anyone come to you to discuss options for spacing births or preventing another pregnancy?** YES NO
- If YES – **tell me about those conversations.** PROCEED TO QUESTION 14

13d If NO to 13c: **Did anyone attend to you at your most recent birth?** YES NO
If YES: **Who attended to you?**

Interviewer - if it was a medical provider, including TBA: **At that time, did the provider approach you to discuss options for spacing births or preventing another pregnancy?** *[tell me about those conversations.]*

14 I'm interested in learning more about your community. Are you aware of which methods, if any, the women in your community use? Please describe. What kinds of women do you think use these methods?
if respondent did not mention natural methods: **What kinds of women do you think use natural methods like withdrawal and the rhythm method to prevent pregnancy?**

15 Do you feel that family planning services are equally available to married and unmarried women in your community? YES NO DK
Why/why not?

16 How satisfied are you with your current method(s) of family planning?
VERY SATISFIED SATISFIED NEITHER UNSATISFIED VERY UNSATISFIED DON'T KNOW.
What kinds of side effects, if any, have you experienced? [wait for answer]
Do you think you will continue to use your current method(s) in the future? YES NO
Modern only: **if your current method were not available to you, what other methods would you consider using?**

16a If WILL NOT CONTINUE USING THIS METHOD: **What other method(s) are you considering?**
Can you tell me some of your reasons for considering that method/those methods.

- *If modern methods:* **Do you know how you would obtain that method/those methods?**

17 I have asked you a lot of questions today about childbearing and pregnancy prevention. Do you have additional thoughts on these matters that you would like to share with me?

Thank you for taking the time to talk with me today.

Note: full sheet rendering of diagram below was given to respondents for questions on scale.

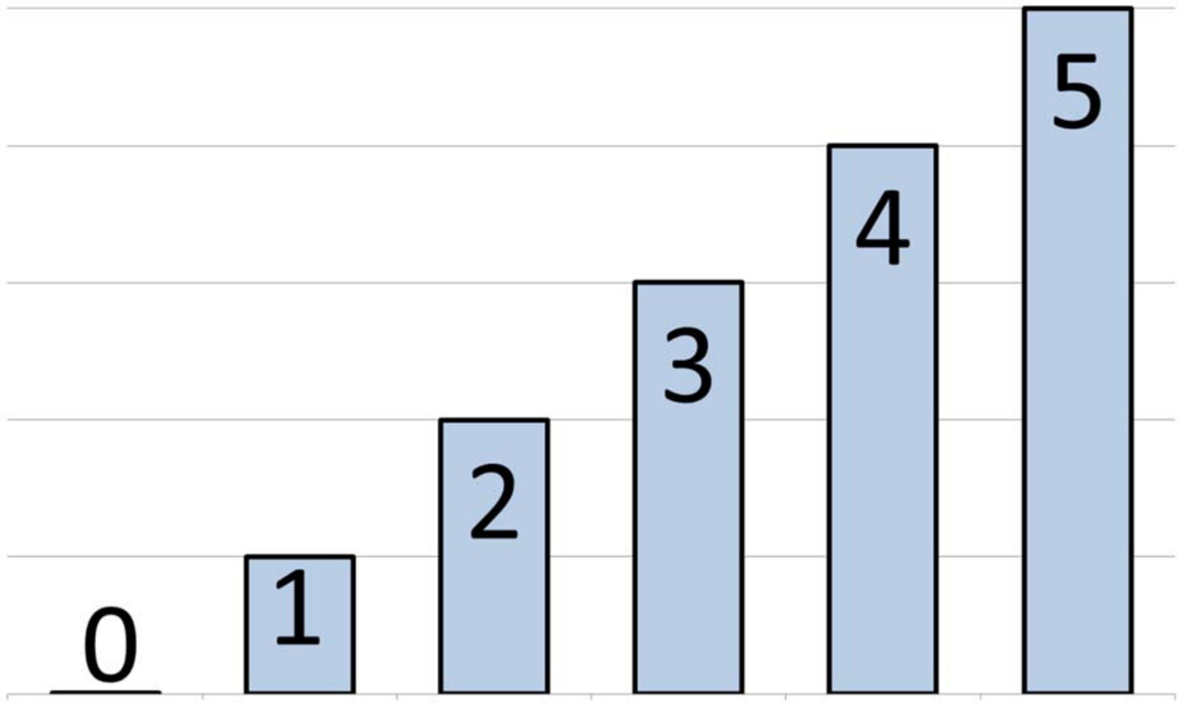

Supplement: S1 File — (PDF) [file pone.0182076.s001.pdf]
